# Severe Burn-Induced Intestinal Epithelial Barrier Dysfunction Is Associated With Endoplasmic Reticulum Stress and Autophagy in Mice

**DOI:** 10.3389/fphys.2018.00441

**Published:** 2018-04-23

**Authors:** Yalan Huang, Yanhai Feng, Yu Wang, Pei Wang, Fengjun Wang, Hui Ren

**Affiliations:** ^1^School of Nursing, Third Military Medical University (Army Medical University), Chongqing, China; ^2^State Key Laboratory of Trauma, Burns and Combined Injury, Institute of Burn Research, Southwest Hospital, Army Medical University, Chongqing, China; ^3^Department of Gastroenterology, Southwest Hospital, Army Medical University, Chongqing, China

**Keywords:** burn, autophagy, endoplasmic reticulum stress, intestinal barrier dysfunction, tight junction

## Abstract

The disruption of intestinal barrier plays a vital role in the pathophysiological changes after severe burn injury, however, the underlying mechanisms are poorly understood. Severe burn causes the disruption of intestinal tight junction (TJ) barrier. Previous studies have shown that endoplasmic reticulum (ER) stress and autophagy are closely associated with the impairment of intestinal mucosa. Thus, we hypothesize that ER stress and autophagy are likely involved in burn injury-induced intestinal epithelial barrier dysfunction. Mice received a 30% total body surface area (TBSA) full-thickness burn, and were sacrificed at 0, 1, 2, 6, 12 and 24 h postburn. The results showed that intestinal permeability was increased significantly after burn injury, accompanied by the damage of mucosa and the alteration of TJ proteins. Severe burn induced ER stress, as indicated by increased intraluminal chaperone binding protein (BIP), CCAAT/enhancer-binding protein homologous protein (CHOP) and inositol-requiring enzyme 1(IRE1)/X-box binding protein 1 splicing (XBP1). Autophagy was activated after burn injury, as evidenced by the increase of autophagy related protein 5 (ATG5), Beclin 1 and LC3II/LC3I ratio and the decrease of p62. Besides, the number of autophagosomes was also increased after burn injury. The levels of p-PI3K(Ser191), p-PI3K(Ser262), p-AKT(Ser473), and p-mTOR were decreased postburn, suggesting that autophagy-related PI3K/AKT/mTOR pathway is involved in the intestinal epithelial barrier dysfunction following severe burn. In summary, severe burn injury induces the ER stress and autophagy in intestinal epithelia, leading to the disruption of intestinal barrier.

## Introduction

The intestinal epithelia form a critical interface between the intestinal mucosa and the luminal environment, providing a barrier against luminal toxins, pathogenic organisms and antigenic molecules. It is well known that the tight junctions (TJs) are junctional complexes composed of numerous transmembrane and cytoplasmic components that form a continuous structure as a barrier around the lateral portion of epithelial cells near the luminal surface. The apical intercellular TJ and its associated proteins, such as zonula occludens (ZOs), occludin and claudins are crucial for the maintenance of the paracellular barrier function (Turner, 2009). Therefore, the changes in the expression and localization of TJ proteins would lead to the paracellular barrier dysfunction in intestinal epithelia.

In the early 1990s, a clinical study demonstrated that intestinal permeability was significantly increased shortly after injury in patients suffering from moderate to major burn injuries (Deitch, 1990). Almost at the same time, an animal study also reported that the intestinal paracellular permeability was significantly increased immediately after injury in guinea pigs with 30% full-thickness burns (Epstein et al., 1991). Afterward, many investigators, including ourselves, have found that severe burn injury directly induces the intestinal TJ barrier dysfunction in both patients and animals, resulting in the increased intestinal permeability, bacterial translocation, systemic inflammatory response syndrome, hypercatabolism, sepsis, and multiple organ dysfunction syndrome (Magnotti and Deitch, 2005; Arrieta et al., 2009; Chen et al., 2012; Carter et al., 2013; Clark et al., 2017). However, the mechanisms of intestinal epithelial barrier dysfunction induced by severe burn injury still remain unclear. Therefore, in order to clinically improve recovery and survival of severe burn victims, it is imperative to get a better understanding of the mechanisms that mediate the intestinal epithelial barrier dysfunction following severe burn injury.

The endoplasmic reticulum (ER) plays pivotal roles in maintaining protein homeostasis, which makes sure that the newly synthesized proteins are correctly folded, assembled and modified (Ron and Walter, 2007). When ER homeostasis is disturbed, an adaptive unfolded protein response (UPR) is elicited to enable the cell to respond to and resolve the ER stress via the activation of three ER membrane resident proteins including inositol-requiring transmembrane kinase endonuclease 1 (IRE1), pancreatic ER kinase (PERK) and activated transcription factor 6 (ATF6). Some secretory cells in the intestine, such as Paneth cells and goblet cells, are particularly susceptible to ER stress and extremely dependent upon a proper functioning UPR to maintain cellular integrity. Recently, it has been reported that ER stress and UPR signaling are associated with the pathogenesis of inflammatory bowel disease (IBD) because that the markers of ER stress in intestinal epithelia are typically increased in patients with active IBD (Zhang and Kaufman, 2008; Kaser and Blumberg, 2009; Negroni et al., 2014). Although the underlying molecular mechanisms are not yet clear, previous studies have shown that severe burn injury causes tissue damages by inducing ER stress in liver, lungs and skeletal muscle (Song et al., 2009, 2014; Dong et al., 2017; Ma et al., 2017). However, little is known about the role of ER stress in meditating the intestinal epithelial barrier dysfunction induced by severe burn injury.

Autophagy, an intracellular degradation pathway, refers to the engulfment and processing of cellular proteins, including damaged organelles and misfolded proteins. Autophagy is a multi-step process including initiation, nucleation, elongation and fusion. And these steps require the coordinated action of a variety of important autophagy-related genes (ATG). It is possible that ER stress can regulate selective autophagy, since the ER is connected with autophagy in various ways, including ER stress-mediated autophagy activation and the formation of autophagosomes at the ER (Rashid et al., 2015). Autophagy occurs at basal level in the normal intestinal mucosa, and can also be induced under the condition of nutrient starvation, hypoxia or ischemia. The previous animal studies have demonstrated that autophagy is remarkably induced in heart, liver, lungs, and intestine following severe burn injury, which in turn contribute to the damages of these visceral organs (Xiao et al., 2012; Song et al., 2014; Dong et al., 2017; Zhang et al., 2017). It has been shown that dysfunctional autophagy leads to chronic intestinal inflammation, and there is a direct relationship between intestinal TJ barrier dysfunction and persistent intestinal inflammation (Cooney et al., 2010; Wildenberg et al., 2012). However, the role of autophagy and ER stress in the regulation of intestinal epithelial TJ barrier remains unknown. Thus, we take the lead in hypothesizing that both ER stress and autophagy are induced in intestinal epithelial cells by severe burn injury, thereby participating in the occurrence and development of the burn-caused intestinal TJ barrier disruption.

## Materials and Methods

### Ethics Statement

The animal studies were approved by the Animal Care and Use Committee of the Third Military Medical University (Army Medical University), and all the protocols were approved by the Ethics Committee of Southwest Hospital, Third Military Medical University (Army Medical University), Chongqing, China.

### Animals Model of Burn Injury

Healthy female C57/BL6 mice (6–8 weeks old), weighing 20–25 g, were purchased from the Animal Center, Third Military Medical University (Army Medical University), and allowed to acclimate for 1 week prior to the experiment. All animals were treated in accordance with the Guide for the Care and Use of Laboratory Animals, and all experiments were approved and performed following the guidelines of Southwest Hospital ethics committee of China. Mice were housed in a temperature-controlled cages with a 12 h light/dark cycle, and allowed access to laboratory chow and water *ad libitum*. Mice were anesthetized with 1 g/L pentobarbital sodium (30 mg/kg) (Xiao et al., 2012). The dorsal and lateral surfaces were shaved. The dorsum was then immersed in 90°C water for 10 s to produce a full-thickness burn with 30% total body surface area (TBSA) (Abdullahi et al., 2014). After injury, lactated Ringer’s solution (1 mL) was administered intraperitoneally for resuscitation. Mice were housed in separate cages and allowed free access to food and water. After the mice woke up from the anesthesia, buprenorphine (2.0 mg/kg) was subcutaneously administered once every 6 h for pain control (Gades et al., 2000). The sham burn mice (0 h) received the same treatments except the temperature of the water was 37°C. At the end of experiment, mice were anesthetized to monitor intestinal permeability, and then sacrificed, followed by tissue harvest for histological, immunofluorescent, and Western blot analysis, respectively, as described below in detail.

### *In Vivo* Intestinal Paracellular Permeability Assay

According to the methods we described previously (Chen et al., 2012), the intestinal paracellular permeability was determined by measuring the appearance in blood of a maker, 4.4 kDa fluorescein isothiocyanate-labeled dextran (FITC-dextran) (Sigma, St. Louis, MO, United States). Briefly, after a laparotomy under anesthesia, a 5-cm segment of the ileum adjacent to the cecum was dissociated, with well-protected superior mesenteric vessels. After the bilateral end of the isolated ileum was ligated with 2-0 silk suture, 0.2 ml of 20 mg/ml FITC-dextran prepared with 0.1 mol/L PBS (pH 7.2) was injected into the lumen. After 30 min, the blood was drawn and centrifuged at 4°C, 3,000 × *g* for 10 min. The plasma was taken and diluted at 1:10 with PBS. Then, the fluorescence intensity of the diluted plasma was measured by a microplate reader (Varioskan Flash, Thermo Electron Corporation, Vantaa, Finland) with an excitation wavelength of 480 nm and an emission wavelength of 520 nm. The concentrations of plasma FITC-dextran were calculated from a standard curve generated by serial dilution of FITC-dextran in PBS.

### Western Blot Analysis for Assessment of Protein Expression

The ileal mucosa scrapings were homogenized in RIPA buffer. The lysate was centrifuged at 15,000 × *g* for 15 min at 4°C and aliquots of supernatants were used for protein expression assessment by Western blot assay. Protein concentrations were determined by *RC DC* kit (Bio-Rad, Hercules, CA, United States), and Laemmli gel loading buffer was added to the lysate and boiled for 5 min. For each lane, 30 mg protein was loaded and separated on 10% SDS-PAGE gel and transferred to PVDF membrane (Millipore). Membranes were blocked using 5% non-fat milk at room temperature for 1 h and then probed with the following primary antibodies at 4°C overnight: anti-ZO-1, anti-occludin, anti-claudin-1 (Invitrogen, Carlsbad, CA, United States), anti-claudin-2, XBP1 (Abcam, Cambridge, MA, United States), CHOP, Bip, PI3 kinase p85, phosphorylated PI3 kinase p85(Tyr458)/p55(Tyr199), Akt, phosphorylated Akt(Ser473), mTOR, phosphorylated mTOR(Ser2448) (Cell Signaling Technology, Danvers, MA, United States), p62, Beclin 1, LC3B, ATG5, β-actin (Sigma). After washing, membranes were incubated with peroxidase-conjugated secondary antibodies (Southern Biotech, Birmingham, AL, United States) for 1 h at room temperature. The blots were visualized with an enhanced chemiluminescence detection kit (GE Healthcare, Buckinghamshire, United Kingdom), and imaged using a ChemiDoc XRS system (Bio-Rad, Hercules, CA, United States). Densitometric analysis was performed using Quantity One software (Bio-Rad).

### Histological Analysis

The ileal tissues were promptly rinsed with cold 0.9% saline solution and immediately fixed in 10% buffered formalin phosphate (pH 7.0) until processing for histological sections. The formalin-fixed samples were embedded in paraffin, and sectioned. After deparaffinization and dehydration, the sections were stained with hematoxylin and eosin for histological assessment of intestinal mucosa. Histological changes of intestinal mucosa were observed with a DM6000B microscope (Leica, Germany), and images were obtained using Metamorph 7.5 software (MDS Analytical Technologies, Downingtown, PA, United States).

### Immunofluorescent Staining, Microscopy, and Image Analysis

Frozen sections of ileal tissue were fixed with 1% paraformaldehyde in PBS containing 1 mmol/L CaCl_2_ for 30 min at room temperature, then washed thrice with PBS for 5 min, permeabilized in 1% Triton X-100 in PBS at room temperature for 5 min. After washing with PBS, the non-specific binding sites were blocked with 5% normal goat serum in PBS for 30 min. Then, the sections were incubated with monoclonal rabbit antibody against ZO-1, occludin, claudin-1 or claudin-2 (Invitrogen) diluted at 1:200 with 1% normal goat serum in PBS at 4°C overnight. After washing thrice in PBS, sections were incubated with secondary Alexa Fluor 594 goat anti-rabbit IgG antibody (Life Technologies) at 1:200, Alexa Fluor 488-conjugated phalloidin (Invitrogen) at 1:100, and DAPI (Sigma) at 1:1,000 for 1 h at room temperature. After washing thrice in PBS, sections were mounted using Slowfade reagents (Invitrogen). Images were obtained using a TCS SP5 laser confocal microscopy (Leica, Germany).

### Transmission Electron Microscopy

The ileal tissues of mice were promptly fixed with 2.5% glutaraldehyde in 0.1 mol/L PBS (pH 7.4) for 2 h, and electron microscopy was performed as previously described (Yamamoto et al., 1991). Morphometric analysis was performed as previously described (Mizushima et al., 2001).

### Statistical Analysis

SPSS 13.0 software was used for statistical analysis. The statistical differences were performed using the Student *t*-test. Data are presented as mean ± SEM. Significance was accepted at *P* < 0.05.

## Results

### Severe Burn Increases Intestinal Paracellular Permeability

**FIGURE 1:**
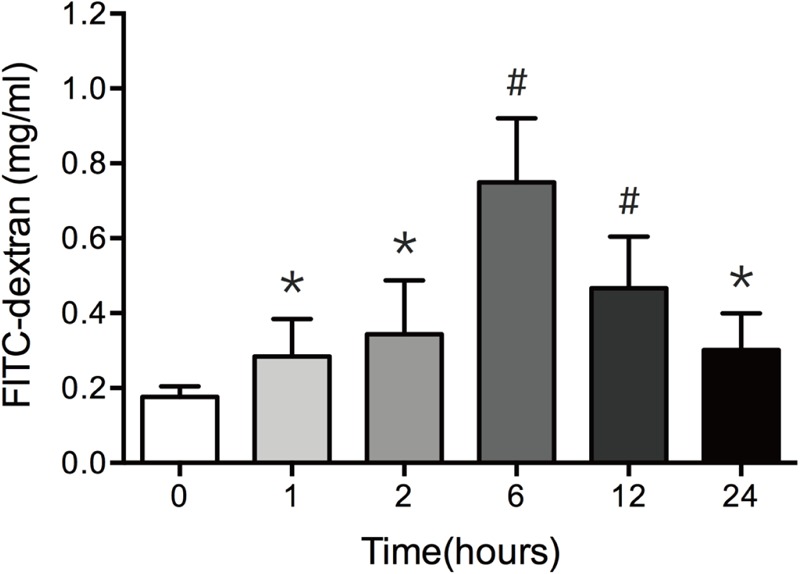
Burn injury increases intestinal permeability. Intestinal permeability was assessed by measuring FITC-dextran in the systemic circulation after intraluminal injection of 4.4-kDa FITC-dextran at 0, 1, 2, 6, 12, and 24 h after 30% TBSA burn injury. Intestinal permeability to 4.4-kDa FITC-dextran was significantly increased after burn injury, *n* = 6–8. Data represent the mean ± SEM. ^∗^*P* < 0.05, ^#^*P* < 0.01 compared with 0 h.

*In vivo* intestinal paracellular permeability was assessed by measuring the concentration of FITC-dextran in the systemic circulation following intraluminal injection of the fluorescent tracer. We did the time-course analysis of intestinal paracellular permeability in mice subjected to 30% TBSA full-thickness burn. As shown in **Figure 1**, the intestinal paracellular permeability to 4.4 kDa FITC-dextran was significantly increased at 1 h following burn injury, and peaked at 6 h with approximately fivefold of 0 h group. This result is similar to our previous report (Chen et al., 2012). Therefore, the increased intestinal paracellular permeability suggests that the severe burn causes the intestinal barrier dysfunction.

### Severe Burn Alters TJ Proteins Expression and ZO-1 Localization

**FIGURE 2:**
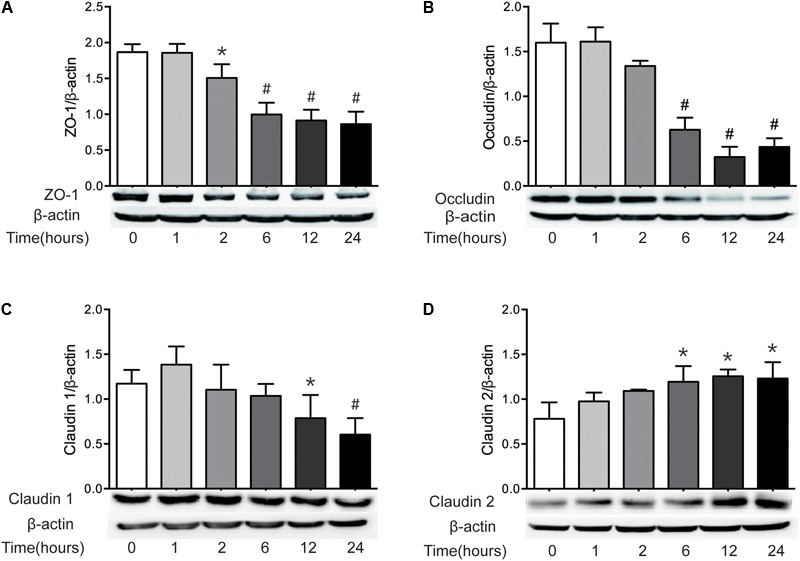
The expressions of TJ proteins are changed markedly after severe burn. The expressions of TJ proteins ZO-1 **(A)**, occludin **(B)**, claudin 1 **(C)**, and claudin 2 **(D)** were analyzed by immunoblot at 0, 1, 2, 6, 12, and 24 h after 30% TBSA burn injury, with β-actin as the loading control, *n* = 6. Data represent the mean ± SEM. ^∗^*P* < 0.05, ^#^*P* < 0.01 compared with 0 h.

It has been well recognized that TJ proteins play critical roles in the regulation of intestinal epithelial barrier function. Previous studies have revealed that the alteration of TJ protein expression is involved in the intestinal barrier disruption (Amasheh et al., 2009; Suzuki et al., 2011). Thus, having found the increase of intestinal permeability following severe burn injury, we then investigated the expression of intestinal epithelial TJ proteins in severely burned mice. As show in in **Figure 2**, the levels of ZO-1 and occludin reduced markedly at 2 h and 6 h following burn injury, respectively (**Figures 2A,B**). The protein level of claudin-1 reduced significantly at 12 h postburn (**Figure 2C**). The protein level of claudin-2, a pore-forming TJ protein, was increased markedly at 6 h after severe burn (**Figure 2D**). Based on these results, it is suggested that the changes of TJ proteins in intestinal epithelia are involved in the intestinal epithelial barrier dysfunction following severe burn.

**FIGURE 3:**
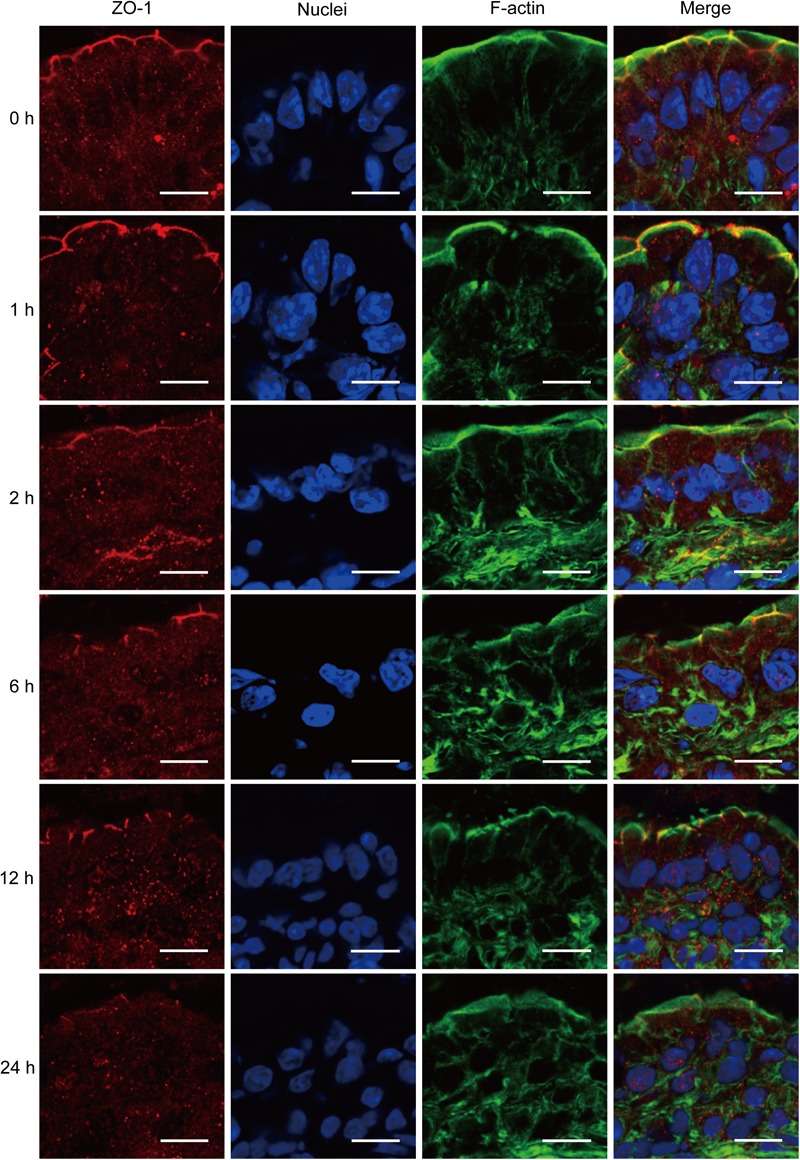
Severe burn injury induces reorganization of tight junction protein ZO-1. Frozen sections of distal ileum were labeled for ZO-1 (red), F-actin (green), and nuclei (blue) at 0, 1, 2, 6, 12, and 24 h after 30% TBSA burns. ZO-1 was localized to the epithelial tight junctions, and colocalized with the perijunctional F-actin ring at 0 h. ZO-1 was stained at the apical junctions in ileum from burned mice, with the loss of ZO-1 at the apical junctions. Data are representative of five independent experiments. Scale bar = 10.0 μm.

ZO-1 has been believed to be the most important TJ protein in the maintenance and regulation of intestinal leaky pathway. Previous studies, including ours, have demonstrated that intestinal epithelial barrier dysfunction is associated with the relocalization of ZO-1 (Fasano, 2011; Chen et al., 2012). Thus, to better understand the intestinal barrier defect induced by severe burn, we further evaluated the morphological changes of ZO-1 protein by immunofluorescent assay. As shown in **Figure 3**, in sham burn mice (0 h), ZO-1 labeled as red was localized to the epithelial tight junctions, and appreciated as a series of bright red spots at the apical compartments of cell–cell junctions. Meanwhile, ZO-1 was colocalized with the perijunctional filamentous (F)-actin ring labeled as green. In contrast, this ordered appearance of ZO-1 was disrupted in burned mice, with the loss of bright red spots representing ZO-1 at the apical junctions. The morphological changes of ZO-1 after severe burn are obvious at 6, 12, and 24 h postburn as compared with 0 h. These results indicate that the intestinal barrier dysfunction is accompanied by the reorganization of TJ protein after severe burn injury.

### Severe Burn Causes Histological Damage of Intestinal Mucosa

**FIGURE 4:**
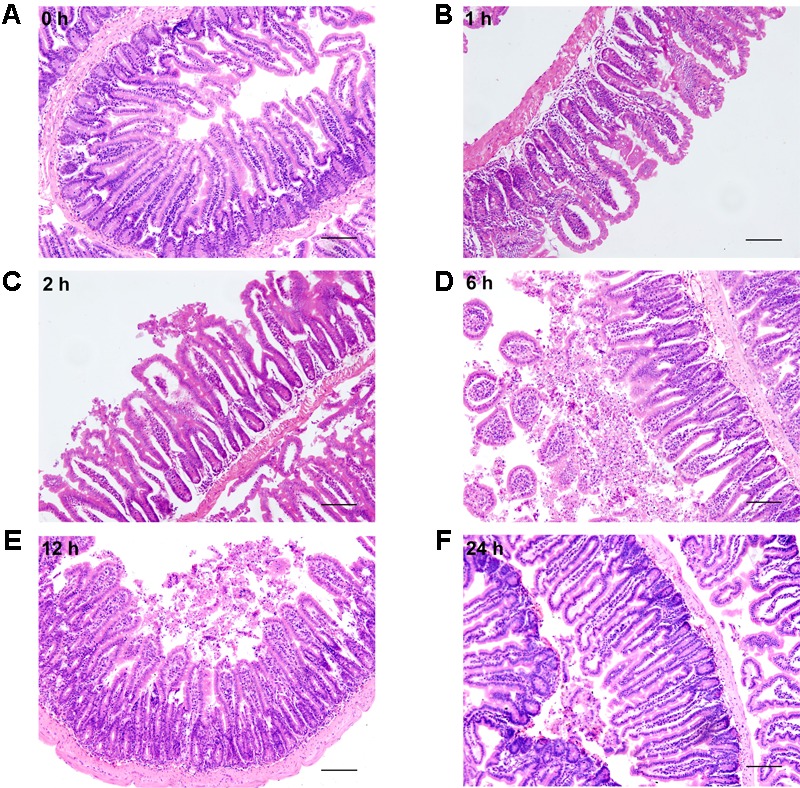
Severe burn injury causes histological damage of intestinal mucosa. Hematoxylin and eosin staining of distal ileal segments was performed at 0, 1, 2, 6, 12, and 24 h following 30% TBSA burns. Normal appearance was shown in the ileum at 0 h **(A)**. Histological damage was characterized by the irregular villi, exfoliated accompanying infiltration of chronic inflammatory cells in the lamina propria, degeneration and necrosis in burned mice **(B–E)**. The damage aggravated with the prolongation of time, and the most severe damage occurred at 6 h postburn **(D)**. The burned mice showed near-normal villi with a few of infiltrating inflammatory cells at 24 h postburn **(F)**. Data are representative of five independent experiments. Scale bar = 200 μm.

Histological examination of distal ileum showed that intestinal villi were in good order and the mucosal epithelia were intact at 0 h (**Figure 4A**). On the contrary, the mucosa was seriously damaged in burned mice. The villi were disorganized, and accompanied by the infiltration of inflammatory cells in the lamina propria at 1 h following severe burn (**Figure 4B**). And with the time prolonged, the more serious damages were observed, such as villous edema at 2 h postburn (**Figure 4C**), exfoliated epithelial cells, and exposed lamina propria, necrosis, as well as signs of inflammatory cell infiltration at 6 h and 12 h postburn (**Figures 4D,E**). However, the villi showed an appearance similar to that of 0 h group at 24 h after burn injury, but still with a few of infiltrating inflammatory cells (**Figure 4F**). These histological features induced by severe burn were consistent with the above-mentioned changes of intestinal paracellular permeability.

### Severe Burn Induces ER Stress in Intestinal Mucosa

**FIGURE 5:**
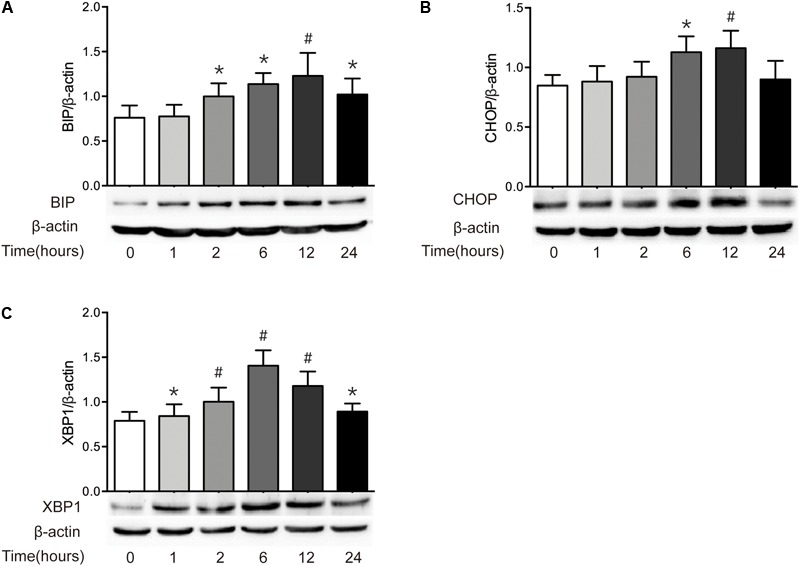
Severe burn induces ER stress in intestinal mucosa. The expressions of BIP **(A)**, CHOP **(B)**, and XBP1 **(C)** in intestinal mucosa were detected by immunoblot at 0, 1, 2, 6, 12, and 24 h following 30% TBSA burn, with β-actin as the loading control. All these proteins were significantly increased after severe burn injury, *n* = 6. Data represent the mean ± SEM. ^∗^*P* < 0.05, ^#^*P* < 0.01 compared with 0 h.

It has been reported that epithelial ER stress contributes to the pathogenesis of intestinal impairment, inducing epithelial cell apoptosis and activating proinflammatory response in the gut (Cao, 2016). Thus, we further detected the level of ER stress in intestine after burn. As illustrated in **Figure 5**, severe burn injury markedly increased the protein expressions of BIP, CHOP, and XBP1. The protein level of BIP, the ER luminal molecular chaperone, was significantly increased at 2 h and peaked at 12 h postburn (**Figure 5A**). CHOP protein markedly increased at 6 h, peaked at 1 h, and returned to 0 h level at 24 h postburn (**Figure 5B**). The protein expression of XBP1 was significantly increased at 1 h, and peaked at 6 h postburn (**Figure 5C**). These findings indicate that an activation of ER stress is induced in intestinal epithelial cells following severe burn injury in mice.

### Severe Burn Causes Autophagy in Intestinal Mucosa

**FIGURE 6:**
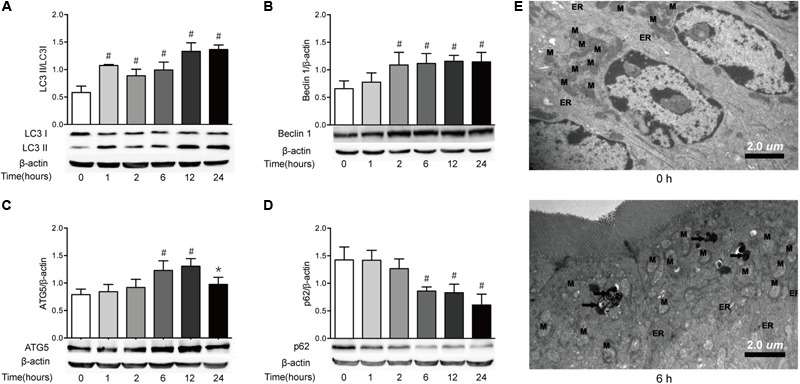
Severe burn induces autophagy in intestinal mucosa. The expressions of LC3B **(A)**, Beclin 1 **(B)**, ATG5 **(C)**, and p62 **(D)** in intestinal mucosa were assayed by immunoblot at 0, 1, 2, 6, 12, and 24 h after 30% TBSA burn injury, with β-actin as the loading control. The expressions of LC3B, Beclin 1, and ATG5 protein were increased following severe burn, whereas the expression of p62 protein was decreased significantly. **(E)** Morphological features of autophagosomes in the intestinal epithelial cells. The structure of intestinal epithelial cells was normal at 0 h. In contrast, the number of lysosomes (black arrow) in the intestinal epithelial cells was increased at 6 h postburn. Data are representative of five independent experiments. ^∗^*P* < 0.05, ^#^*P* < 0.01 compared with 0 h. ER, endoplasmic reticulum; M, mitochondrion. Magnifications: ×8900.

Autophagy can be induced by the increased ER stress, which has been implicated in the pathogenesis of chronic intestinal inflammation (Adolph et al., 2013; Senft and Ronai, 2015). Based on the results that ER stress was enhanced in intestinal epithelial cells after severe burn injury, we then determined the makers of autophagy in ileal mucosa of severely burned mice. The results showed that the expression of LC3, as in the ratio of LC3-II (lipidated form) to LC3-I protein, started to increase significantly at 1 h postburn, and persisted to 24 h (**Figure 6A**). Similarly, the expression of Beclin 1 protein began to increase at 1 h postburn, and maintained a significantly high level of expression until 24 h (**Figure 6B**). Meanwhile, the expression of ATG5 was significantly increased at 6 h postburn, and persisted to 24 h (**Figure 6C**). Consistent with these, the expression of p62 protein was decreased progressively after severe burn injury (**Figure 6D**).

The transmission electron microscopy, which can identify the accumulation of cellular organelles with the morphology of autophagosomes, has been repeatedly reported to be the standard method for monitoring autophagy in tissues (Mizushima et al., 2001). Thus, we also utilized transmission electron microscopy to monitor autophagy in this study (**Figure 6E**). Consistent with the forementioned changes of autophagy markers, no obvious morphological signs of autophagy were observed at the ultrastructural level in the intestinal epithelial cells at 0 h. In contrast, an increased number of lysosomes and the presence of double-membrane structure resembling autophagosomes were observed at 6 h postburn. These indicate that autophagy is induced in intestinal epithelial cells following severe burn injury.

### Severe Burn Downregulates PI3K/AKT/mTOR Pathway in Intestine

**FIGURE 7:**
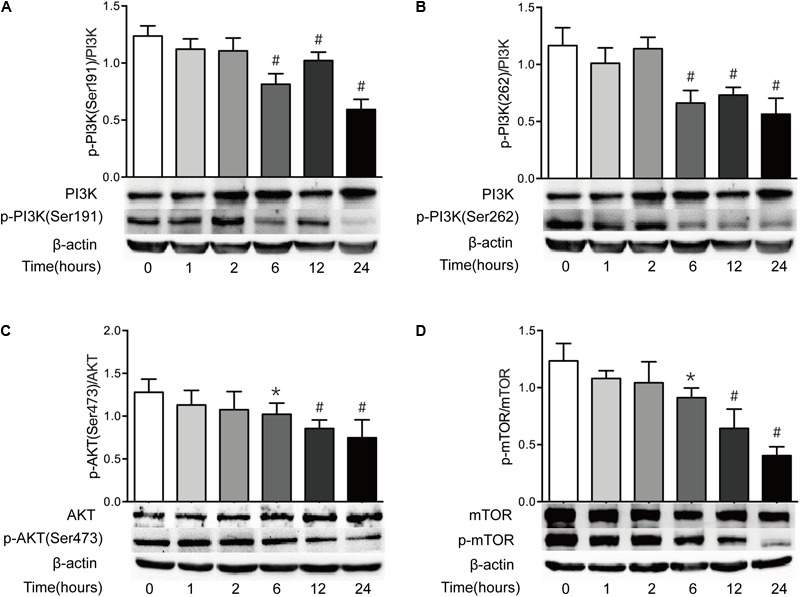
Intestinal PI3K/AKT/mTOR signaling pathway is downregulated after severe burn. The expressions of PI3K/AKT/mTOR signaling pathway-ralated proteins were determined by immunoblot at 0, 1, 2, 6, 12, and 24 h after 30% TBSA burn injury. The ratios of p-PI3K(Ser191)/PI3K **(A)**, p-PI3K(Ser262)/PI3K **(B)**, p-AKT(Ser473)/AKT **(C)**, and p-mTOR/mTOR **(D)** were decreased after severe burn, *n* = 6. Data represent the mean ± SEM. ^∗^*P* < 0.05, ^#^*P* < 0.01 compared with 0 h.

The PI3K/AKT/mTOR pathway is believed to be a central signal transduction pathway in many vital physiological processes, including cell proliferation, apoptosis, morphology, migration, and protein synthesis (Lauring et al., 2013). Previous study has revealed that ER stress leads to the inactivation of the AKT pathway, causing the decrease of mTOR activity and subsequent induction of autophagy (Qin et al., 2010). Having found that both ER stress and autophagy are increased after severe burn injury, we investigated the change of PI3K/AKT/mTOR pathway in this study. As shown in **Figure 7**, the ratio of phosphorylated PI3K at serine191 and serine262 to total PI3K decreased significantly at 6 h postburn, and reached bottom at 24 h (**Figures 7A,B**). Similarly, the ratios of phosphorylated AKT to total AKT and phosphorylated mTOR to total mTOR were progressively reduced, respectively, after burn injury, and reached bottom at 24 h postburn (**Figures 7C,D**). These indicate that PI3K/AKT/mTOR pathway in intestinal epithelial cells is suppressed by severe burn injury.

## Discussion

The intact epithelial barrier is vital for maintaining intestinal homeostasis, and the TJ structure is an essential part of it. The increase of intestinal paracellular permeability has been well recorded in both patients and animals suffering from severe burn injury (Deitch, 1990; Epstein et al., 1991; Magnotti and Deitch, 2005; Arrieta et al., 2009; Chen et al., 2012; Carter et al., 2013). However, it is worth noting that the increase of intestinal paracellular permeability is not specific to burn injury. Other pathological states, such as trauma, systemic ischemia/reperfusion, intestinal ischemia/reperfusion, hemorrhagic shock and systemic inflammation, have also been reported to increase the intestinal paracellular permeability (Langkamp-Henken et al., 1995; Sun et al., 1998; Wattanasirichaigoon et al., 1999; Drewe et al., 2001; Hietbrink et al., 2009). Once the intestinal paracellulare permeability is increased, the luminal antigens and microbes penetrate the defected intestinal epithelial barrier (Ziegler et al., 1988), resulting in the bacterial translocation from intestine to other organs, systemic inflammatory response syndrome, hypermetabolism, sepsis, multiple organ failure. Thus, the consequence of increased intestinal permeability after severe burn injury may lead to a complex response that is closely associated with morbidity and mortality.

Some investigators, including ourselves, have demonstrated that hypoxia and proinflammatory cytokines disrupt intestinal epithelial barrier function, and myosin light chain (MLC) phosphorylation mediated by MLC kinase (MLCK) is involved in the intestinal barrier disruption induced by proinflammatory cytokines or severe burn injury (Wang et al., 2005, 2006; Chen et al., 2012). Although severe burn injury leads to the intestinal barrier disruption, the underlying mechanisms involved in the burn-induced intestinal barrier disruption are still incompletely understood. Recently, increasing studies have revealed that the inductions of both ER stress and autophagy are more closely associated with the pathogenesis of intestinal TJ barrier dysfunction (Zhou, 2011; Elshaer and Begun, 2017). However, little is known about the roles of ER stress and autophagy in the occurrence and development of intestinal TJ barrier disruption after severe burn injury. Therefore, we investigated the paracellular permeability, TJ proteins, ER stress and autophagy in intestinal epithelia after severe burn. Our present study reveals that ER stress and autophagy are involved in the disruption of intestinal TJ barrier after severe burn injury.

The intestinal paracellular permeability is regulated by the TJs located at the apical portion of the epithelial cell–cell contact. It is well known that many critical surgical diseases, such as severe trauma, burn and radiation combined injury, cause the disruption of intestinal epithelial barrier function, allowing the movement of intraluminal contents across the mucosa. In this *in vivo* study, the intestinal epithelial permeability to 4.4 kDa FITC dextran increases significantly at 1 h, peaks at 6 h, and is still higher at 24 h following burn injury. Moreover, the present study also shows that the burn-induced histological damage of intestinal mucosa is significantly aggravating at 6 h and 12 h, and remarkably alleviative at 24 h postburn, which is consistent with the change pattern of intestinal epithelial permeability. Thus, an intact intestinal epithelial barrier function is seriously disrupted after severe burn injury.

The apical TJs are known to have a small-size, cation-selective, high capacity “pore” pathway and a large-size, non-charge-selective “leak” pathway. Both ZO-1 and occludin are very important proteins of TJs and involved in the regulation of leaky pathway. Severe burn injury has previously been shown to increase the intestinal permeability, which is accompanied by the downregulation and altered localization of occludin and ZO-1 (Costantini et al., 2009). Similarly, in this study, the protein expression of both ZO-1 and occludin were markedly reduced, and the localization of ZO-1 was changed obviously after severe burn. Previous studies have demonstrated that claudin-1 is not changed in IBDs (Zeissig et al., 2007). But in this study, the protein expression of claudin-1 is significantly decreased after burn, which is consistent with the previous study revealing that the intestinal barrier defects following severe burn injury is accompanied by the reorganization of TJ proteins ZO-1, occludin and claudin-1 (Chen et al., 2012). It has been shown that claudin-2 expression is increased in the intestinal mucosa in intestinal inflammatory disorders (Zeissig et al., 2007; Costantini et al., 2009), in which the proinflammatory cytokines cause an increase in claudin-2 expression and a claudin-2-dependent increase in TJ permeability (Kinugasa et al., 2000; Heller et al., 2005; Suzuki et al., 2011). Undoubtedly, the increased expression of claudin-2 would cause the intestinal barrier dysfunction, leading to the increase of permeability. Therefore, it is suggested that the altered expression and/or reorganization of TJ proteins may be involved in the intestinal barrier disruption induced by severe burn injury.

Previous studies have reported that defects in epithelial ER stress-related pathways contribute to the pathogenesis of intestinal inflammatory disorders by promoting protein misfolding or by inappropriate UPR responses to normal level, inducing epithelial cell apoptosis and activating proinflammatory response in the gut (Kaser et al., 2010; McGuckin et al., 2010). Under conditions of ER stress, the ER luminal domains of these sensors dissociate from BIP, leading to their activation and the initiation of downstream signaling processes. XBP1 is an important signaling pathway in ER stress. CHOP is a downstream, apoptosis-promoting target of ATF4. CHOP-mediated apoptosis is linked to several ER stress signaling pathways. It has been reported that the XBP1 knockout mice show the increased ER stress, and develop spontaneous intestinal inflammation that strikingly resembles features observed in human IBDs, such as crypt abscesses, leukocyte infiltration and ulcerations, indicating that unabated ER stress in intestinal epithelial cells causes spontaneous intestinal inflammation (Kaser et al., 2008). In the present study, the expressions of XBP1, CHOP, and GRP78/BIP in intestinal epithelial cells are markedly increased after burn injury, indicating that the ER stress is induced by severe burn. The induction of ER stress may contribute to the severe burn-associated intestinal inflammation and barrier dysfunction.

Autophagy, acting as a double-edged sword in cells, means self-eating in charge of maintaining the cell homeostasis. During the induction of autophagy, microtubule associated protein LC3 is lipidated and incorporated into elongating autophagosome, and remains associated with it even after the dissociation of other ATG proteins (Levine and Kroemer, 2008). Since LC3II is converted from LC3I, LC3II/LC3I ratio can sensitively reflect the autophagic status. Beclin 1 is a protein encoded by the Becn1 gene, which is a mammalian ortholog of the yeast A6, and involved in the initiation of autophagosome formation. ATG5 is an essential player in the elongation of the pre-autophagosomal structure. p62, as an autophagy substrate, is an adaptor protein that binds to LC3 and is very important to autophagy under conditions of oxidative stress (Pankiv et al., 2007). It has been reported that starvation-induced autophagy plays critical role in the regulation of intestinal TJ barrier by targeting the degradation of the pore-forming TJ protein claudin-2 in both Caco-2 and MDCK II cells (Nighot et al., 2015). Similarly, other studies have also defined that autophagy is associated with the pathogenesis of Crohn’s disease (Rioux et al., 2007; Groulx et al., 2012). Previous studies have also revealed that autophagy is significantly enhanced in myocardial cells and hepatocytes after severe burn, and that the ER stress in hepatocytes is associated with the sever burn-induced autophagy and liver damage (Xiao et al., 2012; Song et al., 2014). In this study, the autophagy of intestinal epithelial cells is significantly enhanced after severe burn injury, as evidenced by the fact that the LC3II/LC3I ratio, Beclin 1 and ATG5 are significantly increased, and that the expression of p62 is decreased remarkably, accompanied by the occurrence of autophagosomes in intestinal epithelial cells. The enhanced autophagy of intestinal epithelial cells may be one of the players acting in the severe burn-induced intestinal mucosa lesion and barrier disruption.

The mechanisms by which severe burn injury triggers ER stress and autophagy in intestinal epithelial cells are unclear. Previous study has reported that deletion of Xbp1 in Paneth cells results in unresolved ER stress, UPR and induction of autophagy, which can serve as a lesion for the emergence of initiative intestinal inflammation that participates in transmural disease in the absence of the autophagy compensation. It is well recognized that ER stress-induced cell death is mediated by autophagy, which is partly attributed to the inactivation of the mammalian target of rapamycin (mTOR), a central regulator of cell growth that integrates signals from growth factors and nutrients (Ravikumar et al., 2004; Qin et al., 2010; Kim et al., 2011). Interestingly, some studies have revealed that the ER stress downregulates AKT activity, and leads to the suppression of mTOR, thus inducing autophagy (Li and Ren, 2008; Di Nardo et al., 2009). It has been reported that autophagy is induced mainly via PI3K/AKT/mTOR pathway which is playing multiple roles in regulating cell proliferation and differentiation under both physiological and pathological conditions (Aki et al., 2003; Deegan et al., 2013; Ma et al., 2014). Nowadays, it is still controversial about the roles of PI3K/AKT/mTOR signaling pathway in the regulation of autophagy. Some investigators suggest that the activation of PI3K/AKT/mTOR pathway promotes necrotic cell death by inhibition of autophagy (Degenhardt et al., 2006; Wu et al., 2009), while others propose that the activation of PI3K results in autophagic cell death (Aki et al., 2003). In our current study, the ratio of p-PI3K(Ser191)/PI3K, p-PI3K(Ser262)/PI3K, p-AKT(Ser473)/AKT, and p-mTOR/mTOR were all decreased significantly following severe burn, suggesting that severe burn can inhibit the PI3K/AKT/mTOR pathway in intestinal epithelial cells. Thus, it is speculated that the suppressed PI3K/AKT/mTOR signaling pathway may participate in the burn-induced intestinal barrier dysfunction by inducing ER stress and autophagy in intestinal epithelial cells.

## Conclusion

**FIGURE 8:**
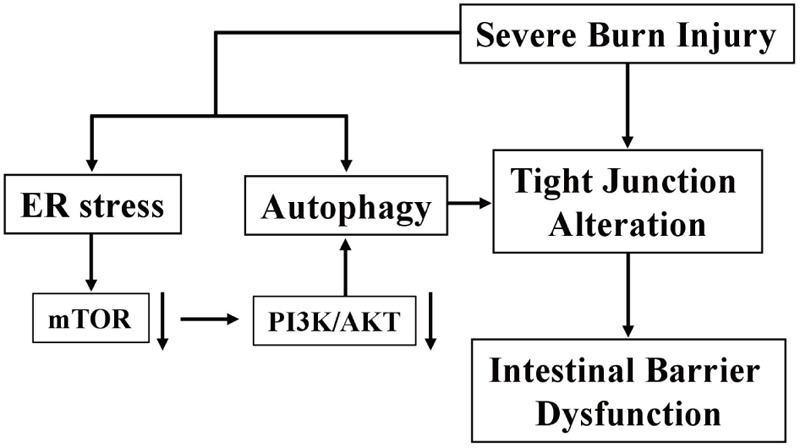
Schematic overview of severe burn-induced intestinal epithelial barrier dysfunction.

In conclusion, severe burn injury induces the disruption of TJ barrier and the increase of intestinal paracellular permeability, which is partly attributed to the enhanced ER stress and autophagy via the inhibition of PI3K/AKT/mTOR signaling pathway in intestinal epithelial cells (**Figure 8**). Our findings are helpful to elucidate the direct and incisive roles of ER stress and autophagy in the severe burn-induced disruption of intestinal TJ barrier, and lighten a new direction to develop novel therapeutic strategies for intestinal barrier dysfunction induced by severe burn injury.

## Author Contributions

YH performed the experiments, drafted the manuscript, and prepared the figures. YF and YW drafted parts of the manuscript and prepared the figures. PW performed parts of the experiments. FW and HR designed the experiments and revised the manuscript.

## Conflict of Interest Statement

The authors declare that the research was conducted in the absence of any commercial or financial relationships that could be construed as a potential conflict of interest.
